# The Effect of Quality Indicators on Beliefs about Medicines Reuse: An Experimental Study

**DOI:** 10.3390/pharmacy9030128

**Published:** 2021-07-21

**Authors:** Yasmin Lam, Rachel McCrindle, Terence K. L. Hui, R. Simon Sherratt, Parastou Donyai

**Affiliations:** 1 Reading School of Pharmacy, University of Reading, Reading RG6 6AP, UK; yasmin.lam@hotmail.co.uk; 2 Department of Biomedical Engineering, School of Biological Sciences, University of Reading, Reading RG6 6AY, UK; r.j.mccrindle@reading.ac.uk (R.M.); t.hui@reading.ac.uk (T.K.L.H.); r.s.sherratt@reading.ac.uk (R.S.S.)

**Keywords:** medicines, sensors, pharmacist, medicines reuse, attitudes

## Abstract

Background: A number of studies have examined beliefs about medicines reuse. Although the practice is prohibited in UK community pharmacy, it does take place elsewhere in the world where it relies on visual checks of returned medicines as an indicator of their quality. One proposal is to integrate sensor technology onto medication packaging as a marker of their quality instead. Our aim was to gauge people’s beliefs about medicines reuse, in an experiment, with or without sensor technology and with or without the promise of visual checks completed by a pharmacist, as experimental conditions, should the practice be sanctioned in the UK in the future. Methods: A between participant study was designed with two independent factors testing the hypothesis that sensors and visual checks would increase pro-medicines-reuse beliefs. A questionnaire was used to measure medicines reuse beliefs and collect qualitative comments. Results: Eighty-one participants took part. Attitudes toward medication offered for reuse, participants’ perceived social pressure to accept the medication, and their intention to take part in medicines reuse all increased with the presence of sensors on packaging and with the promise of pharmacist visual checking, with the former causing a greater increase than the latter, and the combination of both making the greatest increase. People’s qualitative comments explained their concerns about medicines reuse, validating the findings. The use of sensors on medication packaging warrants further investigation if regulators are to consider approving medicines reuse in the UK.

## 1. Introduction

A number of studies have examined people’s views about the idea of medicines reuse, a practice that involves re-dispensing quality-checked, unused, prescribed medication for other patients instead of disposal as waste [1,2,3,4]. This is important because a strong body of evidence shows that inappropriate disposal of unwanted medicines (e.g., disposal via domestic waste and the sewage system), in a host of countries, contributes to the contamination of soil and groundwater with a multitude of drug substances which can even make their way into drinking water [5,6]. Medicines reuse offers a potential solution to minimizing this problem, by encouraging people to return their unwanted medicines to the pharmacy, either for safe disposal or for re-dispensing to other patients [3]. Of course, there are other much more significant ways of reducing environmental contamination from medicinal products, including better research and manufacturing processes at the pharmaceutical industry level, and more responsible prescribing and dispensing practices within the community level [5]. However, medicines reuse, by encouraging people to return their medicines to the pharmacy, also has the potential to help reduce the stockpiling of medicines within patients’ homes, something that can otherwise lead to accidental poisoning and inappropriate self-administration of medicines for undiagnosed conditions [7]. It is also important to remember the financial impact of medication waste, considered in detail elsewhere [8,9]. Finally, there is the potential for medicines reuse to help with the supply of medicines under special circumstances, for example when there are drug shortages or affordability gaps.

Indeed medicines reuse is permitted in some countries such as Greece [10] and the United States [11], mainly for benevolent reasons—the idea being, briefly, that suitable medicines can re-enter the supply chain and be reissued to others in need. However, medicines reuse is prohibited in the UK. The same is true for most other European counties too. A snapshot report on the fate of unused pharmaceuticals in a number of European countries, published in 2013 pointed to a promising recycling scheme in Hungary named Recyclomed [12]. However, on inquiring about the scheme the authors of this paper were recently informed, via email, that for drug safety reasons drug manufacturers opposed both the reuse of medicines as well as any recycling of the packaging via that scheme. Thus, in Hungary, the UK and most other countries in Europe, the main intervention for addressing the inappropriate disposal of unwanted medicines is to offer formal waste collection services for the sole purpose of incineration [12]. The exception is a scheme offered in Portugal named Valormed, which although does not support medicines reuse, does separate collections into elements to be incinerated and packaging components to be recycled [12].

UK officials often cite uncertainties about the quality, safety and efficacy of returned medicines kept in patients’ homes, outside of the formal supply chain, as the reason to oppose the practice of medicines reuse [13,14,15]. These risks are also recognized by members of the public who would not want to receive poor-quality, harmful or incorrect medication as a result of medicines reuse [3]. Thus, the re-use or recycling of another patient’s medicine is not normally recommended by the UK’s Department of Health and Social Care (DHSC). This stance also has to be viewed within the context of a reliable, national healthcare service that has no cause to risk compromising patient safety, theoretically or materially, for any potential benefit that might be gained from sanctioning medicines reuse. However, as we have argued elsewhere, a persistent objection to the idea of medicines reuse is unrealistic because unpredictable events such as pandemics [16] and drug shortages [17] result in temporary U-turns in the UK Government’s position on medicines reuse in any case, but in a space devoid of sufficient investment and research. Thus, a reasonable stance might be to recognize the risks associated with medicines reuse and try to ameliorate these with potential solutions. To this end, we propose a robust mechanism for validating the quality and safety of medicines kept within and outside of the formal supply chain, using the novel ReMINDS (www.reading.ac.uk/ReMINDS, Accessed on 17 July 2021) ecosystem. This system relies on active sensing technologies integrated with the Internet of Things platform to indicate the ‘reusability’ of medicines while interconnecting the relevant stakeholders [18,19]. The availability of such a system should make medicines reuse, whether a temporary measure or not, a much safer practice in the future. Regardless, engaging people in medicines reuse, should the practice be sanctioned by medicines regulators in the future, will still rely on their voluntary participation in such a scheme. For example, the recent proposal to permit care homes and hospices in the UK to draw up standard operating procedures to enable medicines reuse if impacted by shortages due to the pandemic, still required active consent from people affected by such a scheme (donating and receiving) [20].

The studies that have examined people’s views about medicines reuse in countries such as the UK and the Netherlands are clear that the practice would be acceptable to many people provided the quality and safety of the medicines can be guaranteed [1,2,3,4]. For example, in the Netherlands, 61.2% of survey respondents were willing to use medication returned unused to the pharmacy by another patient as long as the quality of these medicines was somehow verified [1]. In the UK too, we found 54.5% of our respondents intended to, wanted to (56.5%) or expected to (56.5%) reuse medication in the future [4]. What-is-more, the findings from our theory-driven study [21] highlight the importance of creating conditions that will illustrate to people that medicines reuse would not expose them to additional medication-related risks [4]. The current way in which returned medicines are screened for reuse relies on the judgement of the pharmacist [22] or another registered health professional [20]. However, as we have argued elsewhere, these methods currently only use history-checking and visual checks, and do not uncover the actual prior storage conditions of returned medicines nor the impact of that storage on the contents within—thus act as proxy measures of quality only. The gap in the market, in our view, is for technologies, for example sensors to measure and track the interaction of the storage conditions (e.g., temperature, light, humidity) with the medicinal pack when kept outside of the formal pharmacy supply chain [18,19]. Sensors that work in this way could reassure people, and reasonably regulators, about the continued quality of reused medicines. Indeed, this is the basis of our proposed ReMINDS ecosystem.

To test these ideas with the public, whose participation in medicines reuse would be key to its success in a hypothesized future scenario, we designed a simple two-factor experiment to gauge people’s responses to the idea of medicines reuse, with or without the presence of a sensor to monitor storage conditions and with or without assurances about a pharmacist’s involvement in visual checking the candidate medicine. We imagined that participants would view medicines reuse more favourably with the presence of a sensor and if given specific information about a candidate medicines having been visually checked by a pharmacist.

## 2. Materials and Methods

### 2.1. Design

A between-participants design was used. One independent variable was the presence of a sensor on the medication box (we chose a standard levothyroxine calendar pack) to monitor the storage environment of the medication presented to the participants. This had two conditions, one condition where the packaging was presented without the sensor and one where it was shown with the sensor accompanied by the researcher reading out the following script: “There is a sensor monitoring the storage conditions of this medication box. This means the temperature and humidity is monitored.” The other independent variable was visual checking. This also consisted of two conditions, one condition where no additional information on visual checking was given and one where additional information was provided, that a pharmacist had been involved in visual checking the medicine, with the following script read out: “The pharmacist has performed quality and safety checks before giving this medication to you”. The combination of the different conditions is shown in Table 1.

The dependent variable was medicines reuse beliefs. This was measured by asking the participants to complete a short questionnaire after being shown the medication box for their specific scenario.

To control for any differences in the participants, the scenarios were allocated at random. In addition, some basic demographic data about the participants were collected in order to check for any substantial differences in the four groups. To control for potential experimenter effects, the researcher was careful to give the experimental instructions in a standard way each time, not favouring a certain outcome, nor giving away any verbal/non-verbal cues that would unduly influence the participants. To control for any other differences in the way the medication was perceived, the boxes of levothyroxine tablets presented were totally identical (apart from the presence of the sensor where appropriate).

### 2.2. Hypothesis

Our hypothesis was that participants would give more positive responses to questions about medicines reuse with the presence of the sensor on the packaging compared to without. We also tested another hypothesis that the addition of a statement about the pharmacist’s involvement in the visual checking of the medication would also result in more positive responses to questions about medicines reuse compared to without.

### 2.3. Materials

**Figure 1:**
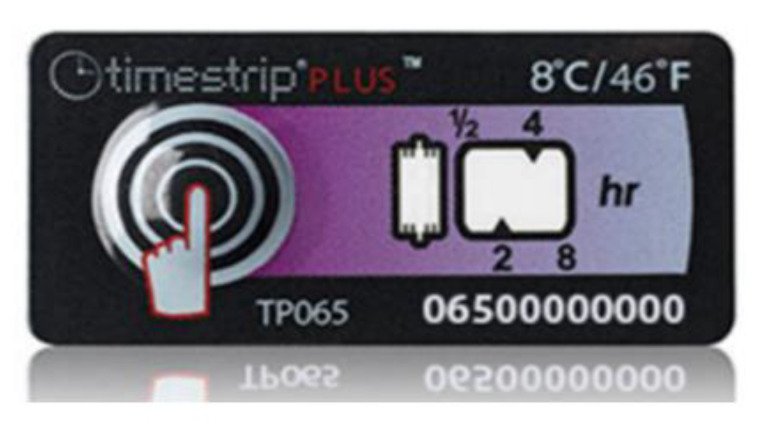
Timestrip^®^ Plus temperature indicator (available from https://timestrip.com/ accessed on 17 July 2021).

Two identical calendar packs of levothyroxine 100 mcg tablets, which usually requires storage at room temperature, were used to represent the medication under consideration. The box containing the ‘sensor’ (used in Experiments 2 and 4) was fitted with a temperature indicator which was a Timestrip^®^ Plus sticker (Figure 1) and a photo-reduced version of an SCS humidity indicator (available from https://staticcontrol.descoindustries.com/ Accessed on 17 July 2021). Both indicators were non-functioning and used for theoretical purposes in order to simulate the monitoring of temperature and humidity, respectively, via a sensor.

**Figure 2:**
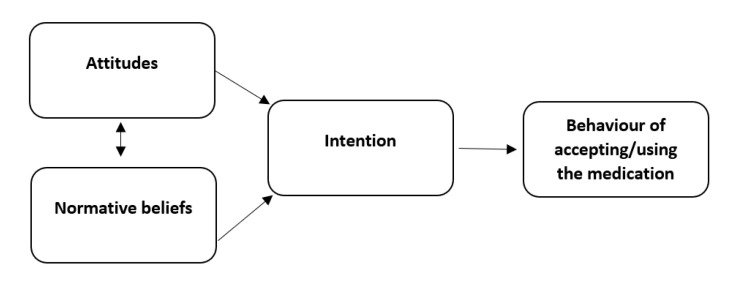
The theory of planned behavior model applied to reusing medication.

The questionnaire was based on an existing, already-validated medicines reuse questionnaire developed using Ajzen’s theory of planned behaviour (TBP) model [23], published fully elsewhere [4] (see Figure 2).

The questionnaire composed of three sections. Section 1 consisted of demographic questions on gender, age, level of education and ethnicity. Section 2 listed 11 statements, each with a five-point Likert response scale to indicate agreement/disagreement with the statements, from strongly disagree to strongly agree. The statements were categorised into three clusters: (a) attitude toward reusing the medicine; (b) normative beliefs/social pressure to accept the medication for reuse; (c) intention to accept the medication (see Table 2). A third section invited the participants to add any comments by hand.

### 2.4. Procedure

Participants from the University of Reading’s Whiteknights Campus were approached and consented via an information leaflet. Each participant was shown a standard empty levothyroxine medication box and allocated at random to receive one of four scenarios all with the same information about medicines reuse; with or without the presence of the sensor, and with or without the visual-check information involving the pharmacist (see Table 1). At the experiment’s start, a standard script was followed for all of the participants:

“This research project is about re-using medicines, which is when a pharmacist gives medication that has been brought back to their pharmacy by one patient, for another patient to use. Currently, in the UK this is not allowed. Imagine you need to collect some medication for yourself from the pharmacy. You are told that the pharmacist had previously given this particular box of medication to another patient (shows participants the medication box, according to scenario and reads out the additional information for Scenarios 2, 3, and 4 as appropriate). That patient did not need this box of medication, so they returned it to the pharmacy a month later. The box is unopened, is the original packaging and the anti-tampering sticker is still attached to it. The medication is also well within the expiry date. This box of medication is then given to you for your own use. Do you have any questions so far? Based on this information, please fill out the questionnaire.”

All participants then completed the questionnaire. The participants were finally debriefed about the aims of the study and asked if they had any questions.

Data from questionnaires were transferred to SPSS^®^ (version 25). The scores from the negatively phrased statements (2, 3) were reversed. The data were then analysed using analysis of variance (ANOVA) with the mean attitude toward the medication, mean perceived social pressure (normative beliefs) to accept the medication, and mean intention to accept the medication, as the dependent variables. The presence of the sensor and inclusion of pharmacist visual-check information were the independent variables.

### 2.5. Participants

Eighty-one participants took part in this experiment. Participants were either staff or students present on the Whiteknights Campus of the University of Reading in November or December 2019. They were approached opportunistically and recruited with an information letter and consent form. There were 41 females, 39 males and 1 who preferred not to disclose their gender. The participants were aged between 18 and 64. There was an even distribution of gender, age, educational qualification and ethnicity across the different experimental scenarios as shown in Table 3.

Forty participants received a scenario (2,4) where the sensor was attached to the packaging and 41 received a scenario (3,4) which informed them that a pharmacist had completed visual checks on the product.

### 2.6. Qualitative Analysis

The qualitative comments left on the questionnaires were also analysed using thematic analysis [24]. This approach was used because it provided a way of organising the qualitative data in the form of themes: recurrent topics, ideas or statements identified across the corpus of data. The comments were analysed manually by YL in consultation with PD, according to the six phases described by Braun and Clarke [24]. The process involved familiarisation with the data, coding, searching for themes, reviewing themes, defining and naming themes, and writing up. After familiarisation with the data, YL coded each comment and assigned initial ‘code names’. The codes were then grouped together under common themes and the themes in turn were grouped according to two higher-order categories.

## 3. Results

**Figure 3:**
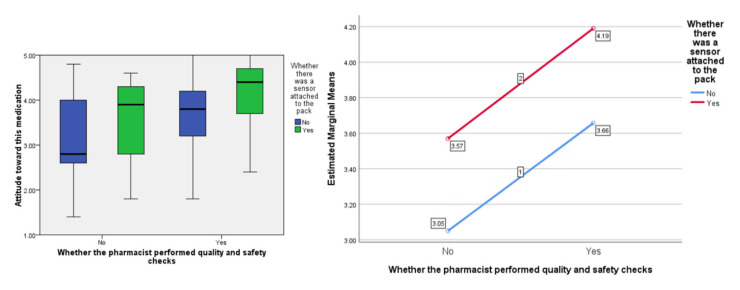
Box plot showing the effect of the independent variables on the ‘attitude’ score (highlighting median values) and, separately, the estimated marginal means plotted against each other to show any interaction (none in this instance).

In terms of people’s ‘attitude’ toward the medication, the F ratios were calculated to be as follows; for the effect of the presence of the ‘sensor’, F (1, 77) = 7.09, *p* < 0.01, the provision of information about visual-checking by the ‘pharmacist’, F (1, 77) = 9.63, *p* < 0.005, and the interaction between sensor and pharmacist, F (1, 77) = 0.001, *p* = 0.974 (see Figure 3).

**Figure 4:**
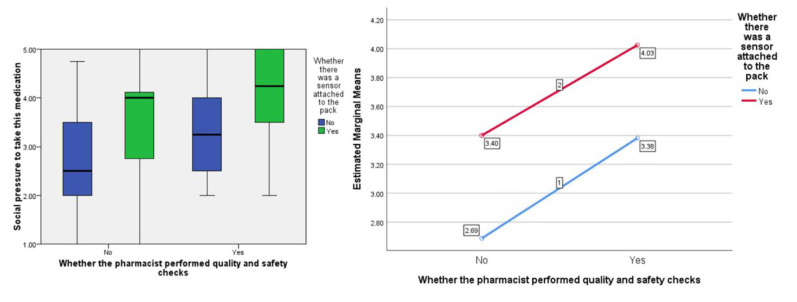
Box plot showing the effect of the independent variables on the ‘social pressure’ score (highlighting median values) and, separately, the estimated marginal means plotted against each other to show any interaction (none in this instance).

In terms of people’s perceived ‘social pressure’ to accept the medication, the F ratios were calculated to be as follows; for the effect of the presence of the ‘sensor’ F (1, 77) = 7.99, *p* < 0.01, the provision of information about visual-checking by the ‘pharmacist’, F (1, 77) = 7.55, *p* < 0.01, and the interaction between sensor and pharmacist F (1, 77) = 0.02, *p* = 0.887 (see Figure 4).

**Figure 5:**
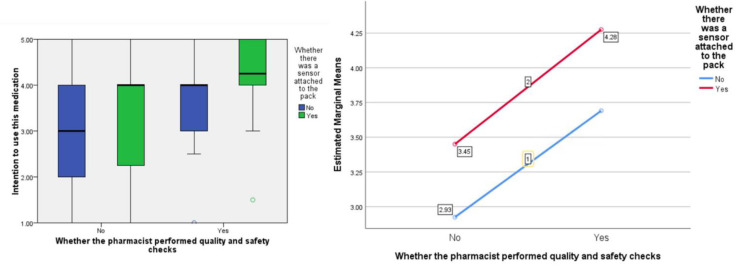
Box plot showing the effect of the independent variables on the ‘intention’ score (highlighting median values) and, separately, the estimated marginal means plotted against each other to show any interaction (none in this instance).

In terms of people’s ‘intention’ to accept the medication, the F ratios were calculated to be as follows; for the effect of the presence of the ‘sensor’, F (1, 77) = 5.21, *p* < 0.05 the provision of information about visual-checking by the ‘pharmacist’, F (1, 77) = 10.71, *p* < 0.005, and the interaction between sensor and pharmacist, F (1, 77) = 0.018, *p* = 0.903 (see Figure 5).

### 3.1. Qualitative Comments

The two super-ordinate categories identified are shown below along with their themes.

### 3.2. Participants’ Expectations of Medicines Reuse

The four themes within this category relate to people’s expectations of medicines reuse.

#### 3.2.1. Physical Characteristics of Re-Dispensed Medicines

Regardless of the scenario, participants wondered whether the medication packaging had been previously opened and wanted factors such as anti-tampering stickers and pharmacist checks to improve their confidence about the authenticity of any reused medication. For example:

- “If you told me it’s been unopened and you can tell by the antitampering sticker, it is fine to take.” (45–54-year-old male, Scenario 1)
- “If tampered then I would give more uncertainty but if checks are in place and come back fine, I would have no issue. (18–24-year-old female, Scenario 4)

#### 3.2.2. Process of Checking for Quality

Participants in Scenarios 3 and 4 were told that a pharmacist had checked the medication for quality and safety. Many thought that a pharmacist was the right professional to be trusted to perform quality and safety checks of returned medicines. Moreover, for some participants in Scenario 4, the presence of a sensor was thought to enhance the pharmacist’s ability to confirm the safety of the medicine. For example:

- It is safe to take as long as pharmacist has checked it (18–24-year-old female, Scenario 3).
- Happier to receive re-used medication that contains a sensor than without due to it aiding the pharmacist with their safety checks. (18–24-year-old male, Scenario 4).

#### 3.2.3. Logistics of Medicines Reuse

In Scenarios 3 and 4, some participants were concerned about the potential burden on pharmacists performing the quality and safety checks of returned medicines due to the additional workload that would be involved. A number of participants in Scenario 3 were uncertain about effectiveness of the checks performed by the pharmacist. For example:

- In theory would reduce medicines wastage however safety cannot be guaranteed even with checks and would be an extra workload on community pharmacists. (25–34-year-old female, Scenario 3).

#### 3.2.4. Incentives to Engage in Medicines Reuse

A number of participants would only engage in medicines reuse under specific conditions. For example:

- I would only take it if there was an incentive e.g., no others available and if it was cheaper/free of charge. (18–24-year-old male, Scenario 1).
- If I was in desperate need of the medication, I would believe that it was safe for me. But I would still be a little cautious when taking it. People can open and re-seal. (18–24-year-old female, Scenario 4).

### 3.3. Understanding the Consequences of Medicines Reuse

#### 3.3.1. Potential Disadvantages of Re-Dispensing Medicines

Some participants expressed uncertainties over the quality and safety of medicines, once handled by other people. Further, some expressed that re-dispensed medicines could be unsafe due to contamination. For example:

- I don not trust where a stranger has put the box. It could change the medication quality. (18–24-year-old, female, Scenario 1).
- I do not like the idea of someone else other than a healthcare practitioner being in handle of my medication. (18–24-year-old female, Scenario 2).
- The packaging could be infected with unidentified bacteria, so taking a risk could be potentially dangerous. (18–24-year-old, Scenario 3).

#### 3.3.2. Potential Advantages of Medicines Reuse

Some participants also mentioned the economic and environmental benefits of reusing medicines. Several participants stated that medicines reuse could aid the NHS in minimizing costs. For example:

- I think if it’s safe and good to use and it would help reduce cost to the NHS. (35–44-year-old female, Scenario 4).
- I think this is a very good idea and will stop wasting the medication. (55–64-year-old female, Scenario 3).
- Re-using unopened medication is a good idea. It could help prevent a shortage of specific medications that are needed. (18–24-year-old female, Scenario 4).

## 4. Discussion

As hypothesized, participants gave more positive responses to questions about medicines reuse with the presence of the sensor on the packaging compared to without. This was across all three domains of attitude toward the medication, social pressure to accept it, and intention to do so. Participants also gave more favourable responses on hearing about the pharmacist’s involvement in the visual checking of the medication compared to without. What-is-more, consistently, the inclusion of the sensor on the packaging resulted in better (more pro-medicines-reuse) responses compared to the visual-checking statement, with the inclusion of both conditions giving the highest scores across attitude toward the medication, social pressure to accept it, and intention to do so. The study provides important evidence about the potential for sensors that measure and track the interaction of the storage conditions with the medicinal pack to reassure people about medicines reuse and encourage them to engage with such a scheme in the future should this be sanctioned by regulators.

A strength of this study is the use of the experimental method. Experiments allow researchers to manipulate the independent variables so that causal inference can be made in terms of the desired outcomes. Thus, through the design of our experiment, we were able to introduce the phenomenon of a sensor and visual-checking information in a controlled manner and then study the impact on people’s pro-medicines-reuse beliefs. This provides a good degree of confidence about the cause-effect of the relationships that we were investigating. A potential weakness is that our participants’ age and education were not representative of the general population or a hypothesized ‘average’ pharmacy customer. While education levels could influence decisions, the likely impact of age on risky decisions is less clear [25]. In addition, because of capacity constraints, each of the four scenarios was tested with only 20 people. Nonetheless, because of the large effect sizes between the different conditions, the study was evidently sufficiently powered to illustrate the statistically significant differences in the outcome measures. Another strength of the study is the accompanying qualitative analysis which provided added explanation to bolster the findings.

The comments from the questionnaire related either to people’s expectations about medicines reuse or illustrated their understanding of the consequences of medicines reuse. In this way, the qualitative comments helped validate the main findings as the participants explained their concerns about the quality of re-dispensed medicines and highlighted how a sensor as well as the involvement of the pharmacist might help increase their confidence in such a product. The comments made by the participants also aligned well with other ideas about medicines reuse found in research elsewhere [1,3,4], for example its role in waste prevention, medicines shortages, its potential impact on pharmacist time and the idea of patient incentives to engage with the practice. One of the most significant outcomes of the current study, however, is the findings that the presence of sensors has a greater impact on people’s pro-medicines reuse beliefs compared with the provision of information about pharmacist checks. Although it makes logical sense to trust technology that can provide objective proof about the storage conditions of medication, over and above the visual checks that a pharmacist might provide, this study adds evidence to the ideas proposed in our ReMINDS ecosystem as a publicly-acceptable solution for reusing returned, prescribed medicines [18,19]. This system relies on active sensing technologies on packaging, integrated with the Internet of Things platform to validate the quality and safety of the medicines while interconnecting the relevant stakeholders [11,12].

Smart packaging concepts are new to medication packs but have been around in the food industry for a number of years [26]. This is not to deny the other sophisticated features of pharmaceutical packaging, which is advanced and well researched [27]. However, the use of technology to enable reuse of medicines is not common and the corresponding research is not at all mainstream [18]. It is important to highlight that smart packaging in that industry consists of more than temperature/humidity sensors, extending to such things as integrity indicators, freshness indicators, and even radiofrequency identification (RFID) tags to identify and locate the product [28]. The current paper only tested the idea of one type of sensor, in a small experiment. Another learning point from the food industry is to consider the environmental impact of the packaging itself against the potential for it to reduce product waste [29]. Therefore, the attachment of sensor technology to medication packaging will not necessarily solve the overall problem of waste created by medication, unless shown to be carbon neutral. While the current paper makes a small contribution to understanding the public’s attitude towards medicines reuse, research on smart packaging within the food industry also offers a wealth of more nuanced information about the impact of such technology on consumer perceptions [30]. For example, a recent review in the food industry unearths not only the functional value of smart packaging (e.g., protecting the content) but also communication value (e.g., perception of safer product), social value (e.g., societal trends towards sustainable living), emotional value (e.g., feeling more confident about the product), and so on [30]. There can also be barriers to the use of smart packaging, such as value barrier (e.g., increased price of final product) and tradition barrier (e.g., getting used to a new type of behavior) discussed in detail elsewhere [30].

It is worth noting that the sensors attached to the medication box (Timestrip^®^ Plus sticker and a downsized version of an SCS humidity indicator) used in the experiments were non-functioning and used for theoretical purposes only in order to mimic the monitoring of temperature and humidity, respectively. More investigations should be undertaken in the future with appropriate indicators, specifically designed to function for medicines reuse schemes. These studies could probe consumer responses in more detail, to examine other potential values and barriers to the use of smart packaging for medication packs.

## 5. Conclusions

This study suggests that the addition of sensors to the packaging of medicines combined with visual quality and safety checks carried out by pharmacists create a more positive response about medicines reuse, compared to their absence. The use of sensors on medication packaging forms the basis of our proposed ReMINDS ecosystem, which warrants further investigation.

## Figures and Tables

**Table 1:** The experimental combinations.

Experiment	Sensor on Packaging	Pharmacist Visual Check
Scenario 1	No	No
Scenario 2	Yes	No
Scenario 3	No	Yes
Scenario 4	Yes	Yes

**Table 2:** The 11 statements categorized according to the theory of planned behaviour (TPB) model. For the analysis, the mean score for ‘attitude’ for each individual was calculated from responses to Questions 1–5, for ‘normative beliefs/social pressure’ from Questions 6–9, and for ‘intention’ from Questions 10 and 11.

TPB Category	Statement
Attitude toward reusing the medication	1. This medication would be safe to use 2. This medication would be harmful 3. This medication would be of low quality 4. This medication would be good 5. It would be satisfying to accept this medication
Normative beliefs/social pressure to accept the medication for reuse	6. My family would believe I should accept this medication 7. My close friends would believe I should accept this medication 8. My doctor would believe that I should accept this medication 9. My pharmacist would believe that I should accept this medication
Intention to accept the medication	10. I would accept this medication 11. I would want to use this medication

**Table 3:** The socio-demographic information of the participants according to the experimental scenarios.

Characteristics	Scenario 1 (*n* = 20) (%)	Scenario 2 (*n* = 20) (%)	Scenario 3 (*n* = 21) (%)	Scenario 4 (*n* = 20) (%)	Total (*n* = 81) (%)
*Gender*					
Female	11 (55.0)	10 (50.0)	12 (57.0)	8 (40.0)	41 (50.6)
Male	9 (45.0)	10 (50.0)	8 (38.0)	12 (60.0)	39 (48.1)
Prefer not to say	0 (0.0)	0 (0.0)	1 (5.0)	0 (0.0)	1 (1.2)
*Age*					
18–24	12 (60.0)	7 (35.0)	11 (52.0)	9 (45.0)	39 (48.1)
25–34	4 (20.0)	9 (45.0)	5 (24.0)	4 (20.0)	22 (27.2)
35–44	2 (10.0)	1 (5.0)	1 (5.0)	5 (25.0)	9 (11.1)
45–54	1 (5.0)	1 (5.0)	3 (14.0)	1 (5.0)	6 (7.4)
55–64	1 (5.0)	2 (10.0)	1 (5.0)	1 (5.0)	5 (6.2)
*Highest qualification*					
GCSE	1 (5.0)	3 (15.0)	4 (19.0)	4 (20.0)	12 (14.8)
A level	8 (40.0)	3 (15.0)	4 (19.0)	7 (35.0)	22 (27.2)
Bachelor’s degree	7 (35.0)	7 (35.0)	7 (33.0)	4 (20.0)	25 (30.9)
Master’s degree	3 (15.0)	2 (10.0)	4 (19.0)	2 (10.0)	11 (13.6)
PhD	1 (5.0)	0 (0.0)	0 (0.0)	2 (10.0)	3 (3.7)
Other	0 (0.0)	4 (20.0)	2 (10.0)	1 (5.0)	7 (8.6)
Prefer not to say	0 (0.0)	1 (5.0)	0 (0.0)	0 (0.0)	1 (1.2)
*Ethnicity*					
English/Welsh/Scottish	8 (40.0)	9 (45.0)	12 (57.0)	8 (40.0)	37 (45.7)
Any other white background	4 (20.0)	0 (0.0)	0 (0.0)	2 (10.0)	6 (7.4)
White and black Caribbean	0 (0.0)	1 (5.0)	0 (0.0)	2 (10.0)	3 (3.7)
White and Asian	0 (0.0)	0 (0.0)	1 (5.0)	0 (0.0)	1 (1.2)
Other mixed	0 (0.0)	0 (0.0)	2 (9.0)	0 (0.0)	2 (2.6)
Indian	1 (5.0)	3 (15.0)	1 (5.0)	0 (0.0)	5 (6.2)
Pakistani	0 (0.0)	2 (10.0)	3 (14.0)	3 (15.0)	8 (9.9)
Bangladeshi	0 (0.0)	1 (5.0)	0 (0.0)	0 (0.0)	1 (1.2)
Chinese	2 (10.0)	1 (5.0)	0 (0.0)	0 (0.0)	3 (3.7)
Other Asian background	2 (10.0)	0 (0.0)	1 (5.0)	0 (0.0)	3 (3.7)
African	1 (5.0)	2 (10.0)	1 (5.0)	2 (10.0)	6 (7.4)
Caribbean	1 (5.0)	1 (5.0)	0 (0.0)	2 (10.0)	4 (4.9)
Other ethnic group	1 (5.0)	0 (0.0)	0 (0.0)	1 (5.0)	2 (2.5)

## Data Availability

The authors can be emailed for further information about the data.
